# Associations Between Objectively Measured Physical Activity, Body Composition and Sarcopenia: Findings from the Hertfordshire Sarcopenia Study (HSS)

**DOI:** 10.1007/s00223-018-0413-5

**Published:** 2018-03-27

**Authors:** Leo D. Westbury, Richard M. Dodds, Holly E. Syddall, Alicja M. Baczynska, Sarah C. Shaw, Elaine M. Dennison, Helen C. Roberts, Avan Aihie Sayer, Cyrus Cooper, Harnish P. Patel

**Affiliations:** 1 MRC Lifecourse Epidemiology Unit, University Hospital Southampton, Tremona Road, Southampton, SO16 6YD UK; 2 Academic Geriatric Medicine, University of Southampton, Southampton, UK; 3 National Institute for Health Research Southampton, Biomedical Research Centre, University of Southampton and University Hospital Southampton NHS Foundation Trust, Southampton, UK; 4 NIHR Collaboration for Leadership in Applied Health Research and Care: Wessex, University of Southampton, Southampton, UK; 5 AGE Research Group, Institute of Neuroscience, Newcastle, UK; 6 NIHR Newcastle Biomedical Research Centre, Newcastle University and Newcastle Upon Tyne Hospitals NHS Foundation Trust, Newcastle, UK; 7 National Institute for Health Research Musculoskeletal Biomedical Research Unit, University of Oxford, Oxford, UK

**Keywords:** Objectively measured physical activity, Accelerometer, Body composition, Physical performance, Sarcopenia

## Abstract

Regular physical activity (PA) is associated with reduced risk of the development and progression of musculoskeletal, metabolic and vascular disease. However, PA declines with age and this can contribute to multiple adverse outcomes. The aims of this study were to describe the relationship between accelerometer-determined PA, body composition and sarcopenia (the loss of muscle mass and function with age). Seven-day PA was measured using the GENEactiv accelerometer among 32 men and 99 women aged 74–84 years who participated in the Hertfordshire Sarcopenia Study. We measured mean daily acceleration and minutes/day spent in non-sedentary and moderate-to-vigorous physical activity (MVPA) levels. Body composition was measured by dual-energy X-ray absorptiometry, muscle strength by grip dynamometry and function by gait speed. Sarcopenia was defined according to the EWGSOP diagnostic algorithm. Men and women spent a median (inter-quartile range) of 138.8 (82, 217) and 186 (122, 240) minutes/day engaging in non-sedentary activity but only 14.3 (1.8, 30.2) and 9.5 (2.1, 18.6) min in MVPA, respectively. Higher levels of PA were associated with reduced adiposity, faster walking speed and decreased risk of sarcopenia. For example, a standard deviation (SD) increase in mean daily acceleration was associated with an increase in walking speed of 0.25 (95% CI 0.05, 0.45) SDs and a reduction in the risk of sarcopenia of 35% (95% CI 1, 57%) in fully adjusted analyses. PA was not associated with hand grip strength. Community-dwelling older adults in this study were largely sedentary but there was evidence that higher levels of activity were associated with reduced adiposity and improved function. PA at all intensity levels in later life may help maintain physical function and protect against sarcopenia.

**Electronic supplementary material:**

The online version of this article (10.1007/s00223-018-0413-5) contains supplementary material, which is available to authorized users.

## Introduction

Sarcopenia is associated with a broad array of adverse physical, metabolic and health-related changes such as reduced mobility, falls, fractures, diabetes, poorer health-related quality of life and death [1, 2]. Physical activity (PA) is defined as bodily movement produced by the bone/muscle unit that results in energy expenditure [3]. Engagement in regular PA such as walking, cycling, work-related activity, sports, gym work, dancing or gardening has been associated with a reduction in the risk of development, or progression, of cardiovascular and cerebrovascular disease, type II diabetes, cognitive decline, obesity, falls and fractures [4–6]. Other benefits of regular physical activity include a greater sense of well-being and self-esteem [7].

However, physical inactivity and sedentary behaviour are common among older people which can lead to an acceleration in muscle catabolism as well as reduced aerobic capacity. In conjunction with other personal, social and environmental factors (such as access to food and social isolation), a decline in physical activity can create a spiral of further inactivity, muscle loss, weight gain, mobility disability and an increase in cardio-metabolic risk [8, 9]. Therefore, measurement and monitoring of PA and improved understanding of its associations with health outcomes are important to inform public health policy.

Objective measurement of physical activity with accelerometers has gained acceptance as a preferred method for the collection of data on the duration, frequency and intensity of activity [10] and is acceptable for large scale epidemiological studies in older people [4, 11]. Several studies have explored the associations between objectively measured PA and measures of body composition and function [12–16]. For example, higher physical activity and low sedentary activity were associated with lower body mass index (BMI) in a cross-sectional analysis of the LIFE study [12]. Aggio et al. showed that in a cohort of 1286 older men aged 70–92, higher levels of MVPA were associated with reduced risk of sarcopenia as defined by the European Working Group on Sarcopenia in Older People (EWGSOP) but where low muscle mass was defined by lower mid-upper arm circumference (MUAC) [4]. In a study of 636 community-dwelling older men and women 59–73 years, Foong et al. showed that higher intensities of PA were associated with higher lean mass and stronger lower limb strength [11]. Associations between PA and better function have also been observed in older adults in assisted care facilities. For example, better accelerometer-based PA was associated with a better short physical performance battery (SPPB) score, grip strength and faster walk speed in older adults aged over 65 [17]. A recent meta-analysis of 25 studies by Steffl et al. concluded that PA, measured by self-report as well as with accelerometers, was associated with reduced odds of acquiring lower muscle mass or ‘sarcopenia’, in later life [18].

Therefore, our aim for this study was to explore the cross-sectional associations between accelerometer derived PA in relation to body composition and sarcopenia as defined by the EWGSOP. This study benefits from the availability of both objectively measured PA and comprehensive characterisation of muscle mass and function by DXA, grip dynamometry and physical performance measures in healthy community-dwelling older men and women aged 74–84 years.

## Methods

### Participants

**Fig. 1:**
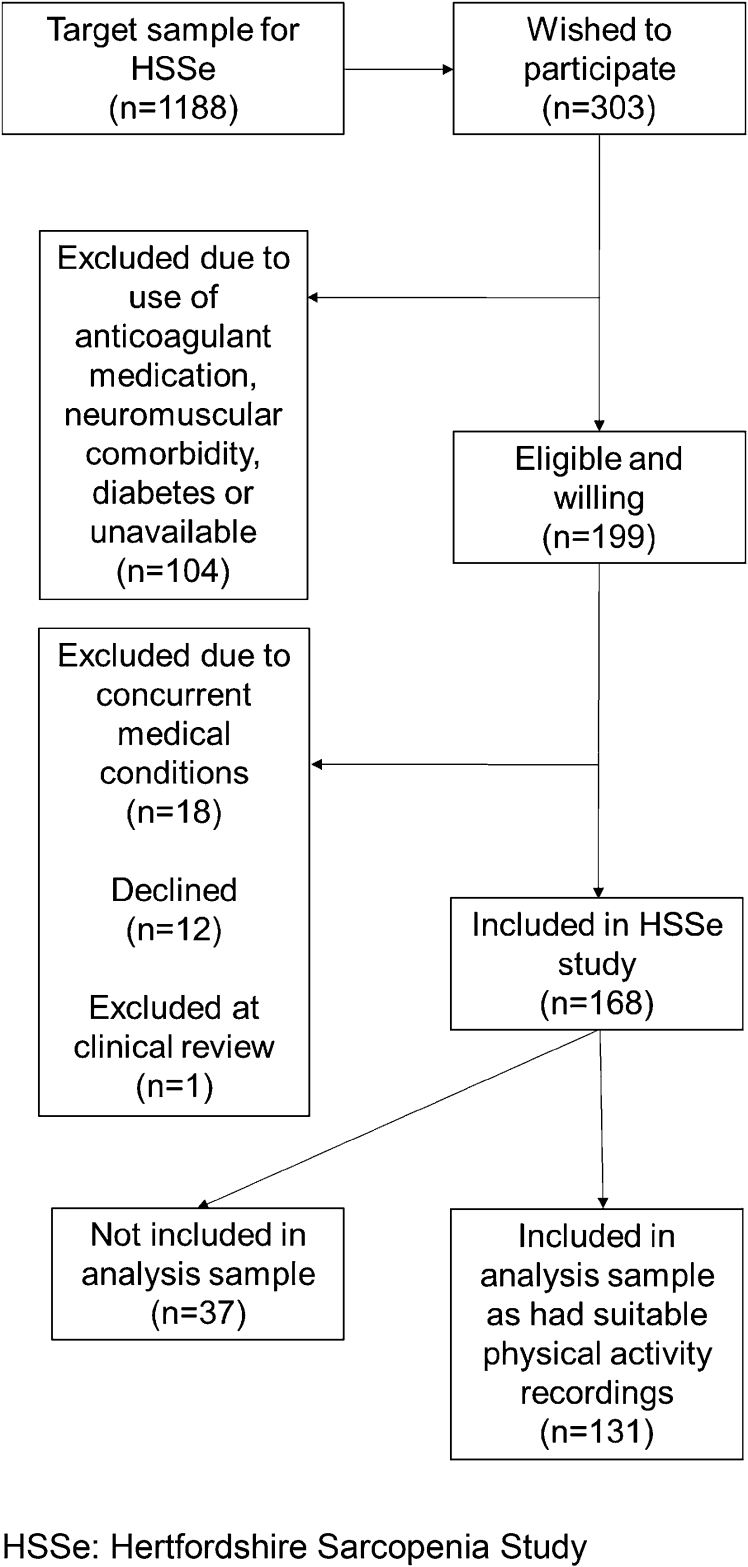
Flow diagram for the analysis sample

The Hertfordshire Sarcopenia Study (HSS) is a retrospective cohort study designed to investigate life course influences on muscle morphology, mass and strength in community-dwelling older people. The first phase of the study focusing on men has previously been described in detail [19]. The second phase of the study (2012–2015) recruited both men and women, herein termed HSSe. Briefly, 1188 participants from the UK Hertfordshire Cohort Study (HCS) [20] were identified as the target sample for HSSe. Of these, 303 wished to participate in the study. All participants were contacted by telephone consecutively and the following clinical exclusion criteria were applied: concurrent use of anticoagulant medication, neuromuscular comorbidity or diabetes. One hundred and ninety-nine participants (66%) were eligible, available and were willing to have a home visit by the research team. Of these, 18 participants were excluded at the time of the home visit due to concurrent medical conditions requiring follow-up and 12 declined to participate further. A total of 169 participants were scheduled to attend a research clinic, but one further participant was excluded at the time of clinical review. Overall, 44 men and 124 women had detailed assessments of body composition and muscle function (Fig. 1).

### Recording of Accelerometry Data

At the time of the home visit, participants were asked to wear a GENEActiv (ActivInsights Ltd, Cambridgeshire, UK) accelerometer on the non-dominant wrist to objectively measure their physical activity. Given limited supply of the accelerometers, devices were activated and posted to participants in some instances. Recordings were made over a 7-day period after the participant started wearing the device. All devices were returned from participants by post. Thereafter, GENEActiv data were downloaded using GENEActiv PC software version 2.2 and saved in raw format as binary files. Accelerometer data were processed in R (http://www.cran.r-project.org) using R-package GGIR [21]. Recording frequencies of 100 and 25 Hz were used.

### Derivation of Objective Physical Activity Measures

A detailed account of the processing of accelerometer data is presented in the supplementary pages online. For each participant, mean daily (over 24-h period) physical activity measures included overall acceleration in milli-g (mg), daily time spent in MVPA (≥ 100 mg) and daily time spent in non-sedentary physical activity levels (≥ 40 mg). The cut-off value for MVPA corresponds to approximately three metabolic equivalents (METs) for adults and has been used in other studies of community-dwelling older people [22, 23]. The range 0–40 mg was chosen as the sedentary category in a previous study involving the use of the GENEActiv accelerometer in older people (average age 79 years) [24]. Overall, 30 participants (18%) were excluded from the analysis for the following reasons: unable to contact; refusal to wear the device; technical problem with the accelerometer or excessive periods of non-wear detected in preliminary analysis. A total of 138 recordings were processed. Of these, two participants had recordings of less than 7 days; one had taken the device off to sleep; and four had unfeasible values and were excluded from analyses. Consequently, the analysis sample comprised 131 participants (32 men, 99 women) with objective physical activity recordings. A flow diagram for the analysis sample is provided in Fig. 1. There were no statistically significant differences regarding the outcome variables of interest between participants with and without suitable physical activity data.

### Measurement of Anthropometry, Physical Performance and Body Composition

Height in centimetres (cm) and weight in kilograms (kg) were measured once. Body composition [total lean mass, appendicular lean mass (ALM), and fat mass] was assessed by dual-energy X-ray absorptiometry (DXA) (Hologic Discovery, software version 12.5) for all participants. Isometric grip strength (kg) was measured three times in each hand using a Jamar handheld hydraulic dynamometer (Promedics, UK) and the maximum value of six measures was used for analysis. Customary walking speed was measured over a three-metre course. Both the DXA machine and dynamometer were calibrated before the study and at regular intervals throughout the study.

### Derivation of Sarcopenia Status

Sarcopenia status at follow-up was derived using the EWGSOP diagnostic algorithm with the following cut-points: appendicular lean mass index (ALM/height^2^) ≤ 7.23 kg/m^2^ for men (≤ 5.67 kg/m^2^ for women); grip strength < 30 kg for men (< 20 kg for women); and walking speed ≤ 0.8 m/s [25]. Participants with slow walking speed or weak grip strength, and who also had low ALM index were classed as having sarcopenia.

### Ascertainment of Socio-demographic and Lifestyle Factors

Social history was ascertained during the HCS baseline interview. At the HSSe interview, smoking status and weekly alcohol consumption were ascertained and details of all prescription and over-the-counter medications currently taken were coded according to the British National Formulary. Medication use across body wide systems (termed number of systems medicated) was used as a marker of comorbidity. We did not ascertain whether our participants were engaged in resistance or endurance-based activity in their leisure time.

### Statistical Methods

Height and weight were highly correlated (*r* = 0.46, *p* < 0.009 for men; *r* = 0.43, *p* < 0.001 for women). To account for multi-collinearity, a sex-specific standardised residual of weight-adjusted-for-height was derived for inclusion with height in regression models. Registrar General’s social class was coded from the 1990 Standard Occupational Classification (SOC90) unit group for occupation using computer-assisted standard occupational coding [26]. Current social class was coded from current or most recent full-time occupation for men and women who never married, and from husband’s occupation for ever-married women.

Data were described using summary statistics. Mean daily time spent in MVPA was skewed and included zeros and was therefore square-root transformed; the other physical activity measures and continuous outcome variables were normally distributed. Associations between physical activity measures were examined using Pearson correlations. Linear regression was used to examine the associations between each mean daily physical activity measure (acceleration, time in non-sedentary levels and time in MVPA levels) and the following outcomes: weight, BMI, total fat mass, ALM index, grip strength and walking speed. Poisson regression models with a robust variance estimator to yield relative risks were used for sarcopenia as the outcome variable. Gender-adjusted and fully adjusted models (accounting for gender, age, height, weight-for-height residual, smoking, alcohol and social class) were implemented. Gender-adjusted and fully adjusted models for ALM index and sarcopenia were adjusted for total fat mass and not for height or weight-for-height residual. Models for weight, BMI and total fat mass were not adjusted for any adiposity measures and models for BMI were also not adjusted for height. Sex-specific standard deviation (SD) scores were coded for physical activity measures and continuous outcomes in models. Due to the small number of men in the sample (*n* = 32), pooled gender-adjusted analyses were conducted. All data were analysed using Stata, release 14.2 (STATA Corp, College Station, TX, USA).

## Results

### Participant Characteristics

The average age at the time of wearing the accelerometer was 79 years. Median daily time spent at non-sedentary activity levels was 138 and 186 min among men and women, respectively. In contrast, the median daily time spent in MVPA was only 14.3 min for men and 9.5 min for women. Overall 5 (16.1%) men and 21 (21.6%) women had sarcopenia (Table 1).

**Table 1:** Participant characteristics

Mean (SD)	Men (*n* = 32)	Women (*n* = 99)
Age (years)	78.6 (2.7)	78.9 (2.3)
Height (cm)	172.4 (6.1)	159.4 (5.9)
Weight (kg)	78.9 (12.2)	70.8 (12.3)
BMI (kg/m^2^)	26.5 (3.7)	27.8 (4.4)
Total body fat mass (kg)	27.1 (9)	31.1 (8.3)
Fat mass index (kg/m^2^)	9.1 (3.0)	12.2 (3.2)
Appendicular lean mass (kg)	21.3 (2.8)	15.2 (2.4)
Appendicular lean mass index (kg/m^2^)	7.2 (0.9)	6.0 (0.8)
Grip strength (kg)	34.8 (6.5)	20.7 (5.6)
Walking speed (m/s)	0.98 (0.18)	0.96 (0.19)
EWGSOP sarcopenia^a^	5 (16.1%)	21 (21.6%)
Ever smoked^a^	14 (43.8%)	38 (38.4%)
Social class (manual)^a^	13 (43.3%)	49 (49.5%)
Ever smoked^b^	14 (43.8%)	38 (38.4%)
Alcohol consumption (units per week)^a^		
Very low (< 1)	7 (21.9%)	55 (55.6%)
Low (1–10 M, 1–7 W)	16 (50%)	32 (32.3%)
Moderate (11–21 M, 8–14 W)	5 (15.6%)	10 (10.1%)
High (> 21 M, > 14 W)	4 (12.5%)	2 (2%)
Acceleration (mg)^+^	23.9 (7.6)	25.5 (6.8)
Non-sedentary (min/day)^b^	137.8 (81.7, 217.2)	186.0 (122.1, 240.4)
MVPA (min/day)^b^	14.3 (1.8, 30.2)	9.5 (2.1, 18.6)

Maximum number of missing observations was nine (total body fat, fat mass index and walking speed)

Manual social class: categories IIIM-V from 1990 OPCS Standard Occupational Classification

Three individuals could not walk without another person’s help and four used a walking aid so their values of walking speed were set to missing

*M* men, *W* women, *MVPA* moderate-to-vigorous physical activity

^+^Average daily (24 h) acceleration for each participant

^a^*n* (%)

^b^Median (lower quartile, upper quartile)

### Associations Between Objective Measures of Physical Activity and Body Composition and Sarcopenia

**Fig. 2:**
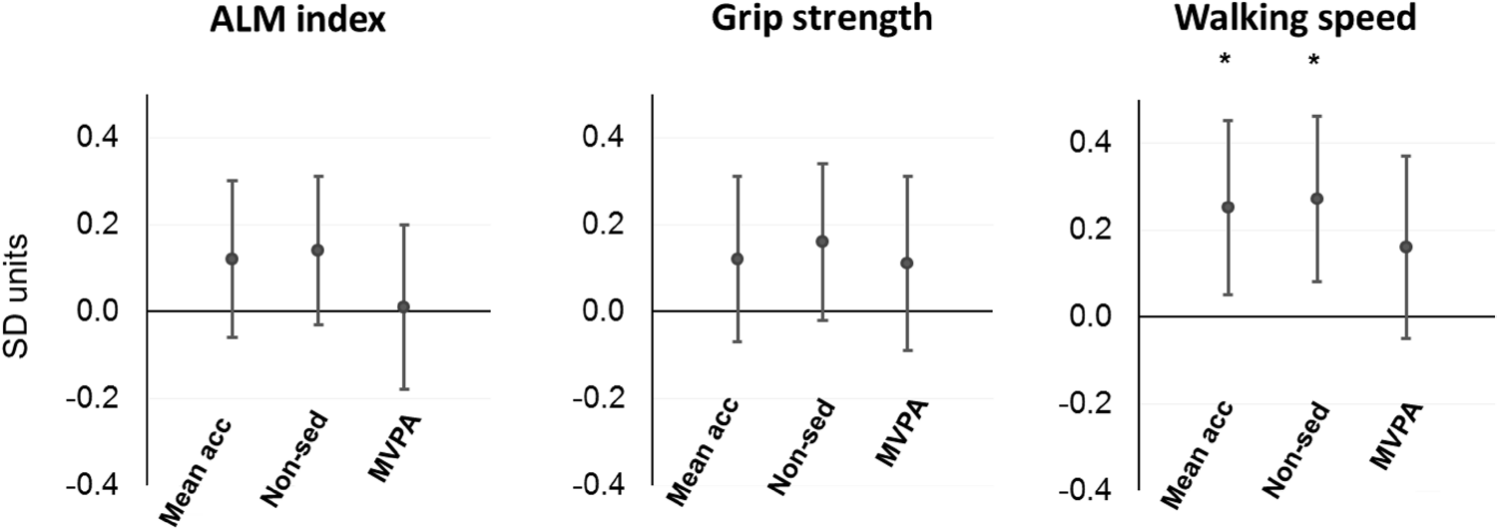
SD difference in outcomes (95% CI) per SD increase in physical activity measures (fully adjusted associations). Estimates are adjusted for gender, age, height, weight-for-height residual, smoking history (ever vs. never), alcohol consumption, and social class (manual vs. non-manual). ALM index was adjusted for total fat mass and not for height or weight-for-height residual. *SD* standard deviation, *ALM* appendicular lean mass, *Mean acc* mean acceleration, *Non-sed* non-sedentary, *MVPA* moderate-to-vigorous physical activity. For each participant, mean daily physical activity measures were derived. *p < 0.05

Mean daily acceleration was strongly correlated with non-sedentary time (*r* = 0.92). Time spent in MVPA was correlated with daily acceleration (*r* = 0.73) and non-sedentary time (*r* = 0.65) (*p* < 0.05 for all correlations). Relationships between physical activity measures and ALM index, grip strength and walking speed are presented in Table 2 as well as Fig. 2. Greater non-sedentary time was weakly associated (*p* = 0.07) with higher ALM index after adjustment for gender and total fat mass but this was attenuated by full adjustment. Greater level of daily acceleration and increased non-sedentary time were associated with faster walking speed in gender-adjusted and fully adjusted analyses. For example, a one SD increase in non-sedentary time was associated with an average increase in walking speed of 0.27 (95% CI 0.08, 0.46) SDs in fully adjusted analyses. None of the physical activity measures were associated with grip strength. Higher physical activity according to each of the three measures was associated with reduced risk of sarcopenia after adjustment for gender and total fat mass (*p* < 0.05); associations regarding mean daily acceleration were robust in fully adjusted analyses (*p* = 0.047). Associations were not substantially altered when additionally adjusted for the number of systems medicated (data not shown).

**Table 2:** SD difference in ALM index, grip strength, walking speed and relative risks for sarcopenia per SD increase in the physical activity measures

Physical activity measure	Outcome	Adjusted for gender		Fully adjusted^a^	
		Estimate (95% CI)	*p* value	Estimate (95% CI)	*p* value
Acceleration	ALM index	0.15 (− 0.03, 0.32)	0.10	0.12 (− 0.07, 0.30)	0.21
	Grip strength	0.11 (− 0.07, 0.28)	0.24	0.12 (− 0.08, 0.31)	0.23
	Walking speed	0.26 (0.08, 0.44)	0.01	0.25 (0.05, 0.45)	0.02
	Sarcopenia	0.67 (0.47, 0.95)	0.03	0.65 (0.43, 0.99)	0.05
Time in non-sedentary levels	ALM index	0.16 (− 0.01, 0.33)	0.07	0.14 (− 0.04, 0.31)	0.13
	Grip strength	0.15 (− 0.02, 0.33)	0.08	0.16 (− 0.03, 0.34)	0.09
	Walking speed	0.29 (0.12, 0.47)	< 0.001	0.27 (0.08, 0.46)	0.01
	Sarcopenia	0.66 (0.45, 0.96)	0.03	0.67 (0.44, 1.01)	0.06
Time in MVPA levels	ALM index	0.06 (− 0.12, 0.24)	0.51	0.01 (− 0.18, 0.20)	0.93
	Grip strength	0.10 (− 0.08, 0.27)	0.29	0.11 (− 0.09, 0.31)	0.27
	Walking speed	0.19 (0.01, 0.37)	0.04	0.16 (− 0.05, 0.37)	0.14
	Sarcopenia	0.65 (0.43, 0.98)	0.04	0.70 (0.43, 1.13)	0.14

For each participant, mean daily physical activity measures were derived

*SD* standard deviation, *ALM* appendicular lean mass

Gender- and fully adjusted models for ALM index and sarcopenia were adjusted for total fat mass and not for height or weight-for-height residual

Estimates for sarcopenia are relative risks obtained from Poisson regression models with a robust variance estimator. All other estimates are regression coefficients obtained from linear regression models

^a^Adjusted for gender, age, height, weight-for-height residual, smoking history (ever vs. never), alcohol consumption, and social class (manual vs. non-manual)

### Associations Between Physical Activity Measures and Weight, BMI and Fat Mass

Associations between physical activity measures and weight, BMI and fat mass are presented in Table 3. In gender-adjusted and fully adjusted analyses, higher physical activity was associated with lower weight, BMI, and fat mass, regardless of the physical activity measure used.

**Table 3:** SD difference in weight, BMI and fat mass per SD increase in the physical activity measures

Physical activity measure	Outcome	Gender-adjusted		Fully adjusted^a^	
		Estimate (95% CI)	*p* value	Estimate (95% CI)	*p* value
Acceleration	Weight	− 0.43 (− 0.58, − 0.27)	< 0.001	− 0.40 (− 0.55, − 0.26)	< 0.001
	BMI	− 0.46 (− 0.61, − 0.30)	< 0.001	− 0.45 (− 0.61, − 0.30)	< 0.001
	Fat mass	− 0.50 (− 0.66, − 0.34)	< 0.001	− 0.49 (− 0.64, − 0.33)	< 0.001
Time in non-sedentary levels	Weight	− 0.35 (− 0.51, − 0.19)	< 0.001	− 0.33 (− 0.48, − 0.18)	< 0.001
	BMI	− 0.39 (− 0.55, − 0.23)	< 0.001	− 0.37 (− 0.54, − 0.21)	< 0.001
	Fat mass	− 0.46 (− 0.62, − 0.30)	< 0.001	− 0.44 (− 0.59, − 0.28)	< 0.001
Time in MVPA levels	Weight	− 0.43 (− 0.58, − 0.27)	< 0.001	− 0.41 (− 0.56, − 0.26)	< 0.001
	BMI	− 0.42 (− 0.58, − 0.26)	< 0.001	− 0.45 (− 0.61, − 0.29)	< 0.001
	Fat mass	− 0.49 (− 0.64, − 0.33)	< 0.001	− 0.51 (− 0.67, − 0.35)	< 0.001

For each participant, mean daily physical activity measures were derived

Models for BMI were not adjusted for height

Estimates are regression coefficients obtained from linear regression models

*SD* standard deviation

^a^Adjusted for gender, age, height, smoking history (ever vs. never), alcohol consumption, and social class (manual vs. non-manual)

## Discussion

In this study of community-dwelling older men and women, we have shown that a higher level of objectively measured PA was associated with reduced adiposity, increased function (faster walking speed) and decreased risk of sarcopenia. PA was not significantly associated with muscle strength or ALM index.

Older men and women in this study only spent a median of 14 and 10 min a day engaging in moderate-to-vigorous physical activity, respectively, falling significantly short of the WHO’s recommendation of at least 150 min of moderate-intensity aerobic physical activity throughout the week for a sustained health benefit [27]. Our results are in accordance with other studies which have shown that older adults engage in significantly fewer minutes of MVPA not only when compared with young adults, but also relative to recommended guidelines [28]. Previous studies of older people have also utilised the threshold of 100 mg to identify MVPA (≥ 3 METS) [22, 23]; however, it should be acknowledged that this threshold was not explicitly developed for older adults [29]. This may explain the low levels of MVPA that were apparent in our study. Importantly however, greater time spent at any non-sedentary level of PA may confer health benefits [30, 31]. For example, a recent systematic review and meta-analysis by Hupin et al. reported that MVPA in adults, even below current recommended levels, conferred a lower mortality risk [32].

Our results regarding associations between PA and adiposity are consistent with the literature. Time spent in non-sedentary activity as well as MVPA has been shown to be associated with lower adiposity and lower BMI in several population-based studies [4, 33, 34] and a systematic review by Fuzeki et al. concluded that engagement in light PA was associated with less risk of developing obesity [35].

Our results regarding the positive associations (*p* < 0.07) observed between PA and ALM index are also in broad agreement with those reported in the literature. For example, Foong et al. showed that among community-dwelling older men and women, light as well as higher intensity levels of PA were positively associated with higher lean mass percentage whilst sedentary activity had the opposite effect [11]. In studies of older Japanese men, moderate or vigorous physical activity (≥ 3 metabolic equivalents [METS]) was associated with lower lean mass index [36], a finding not replicated in our study (although the majority of our sample were female).

We did not see an association between PA and grip strength in our study. Although Gerdhem et al. found no association between accelerometer-based PA and knee muscle strength [37], the large cross-sectional study of older men by Aggio et al. identified positive associations between PA and both grip strength and physical performance [4]. In addition, a large population-based cross-sectional study identified associations between light, moderate and vigorous accelerometer-based physical activity and stronger leg and knee extension strength [11]. Furthermore, sedentary behaviour ascertained by questionnaire analysis was associated with lower grip strength in a study by Hamer and Stamatakis [38]. Other studies have also reported positive associations between objective measures of PA and grip strength [12, 17]. It is possible that HSSe participants may not have been taking part in activities that benefit upper extremity strength. This may provide an explanation for our results [39].

Our finding that increased non-sedentary time was associated with faster gait speed is consistent with previous studies that have reported associations between higher levels of objectively measured light or moderate-to-vigorous PA and better physical performance [40, 41]. Our results suggest an association between higher levels of PA and a lower risk of sarcopenia. In support of our findings, associations between PA and a lower risk of acquiring sarcopenia were confirmed in a recent meta-analysis [18]. To our knowledge, only one previous study has reported an association between higher MVPA and reduced risk of developing severe sarcopenia according to the EWGSOP diagnostic algorithm [4].

Our study has some limitations. First, a healthy responder bias has been observed in HCS and is also evident in HSSe. However, as our analyses were internal, unless there was a systematic difference in the observations of interest among our study participants and those who were invited to take part in the study but did not, no major bias should have occurred. Second, we have a relatively small sample size with no ethnic diversity but this was the largest available; higher attrition from the target sample was expected due to the commitment required from participants to wear the device for 7 days. Third, a cross-sectional study design precludes any judgment regarding causality. Fourth, participants wore the accelerometer during different months of the year which may have influenced their physical activity levels.

Our study also has many strengths. First, the HSSe has a comprehensive set of muscle outcomes (muscle mass and body composition, strength and function) making this relatively unusual among other studies. The study was conducted according to strict protocols by an experienced multi-disciplinary research team. Second, we have obtained objective measures of PA. The GENEActiv and other wearable accelerometers accurately discriminate between different PA intensities and are acceptable and feasible to use in cohorts of older people as they are non-intrusive, waterproof, robust and importantly store substantial amounts of data [42]. In addition, the high criterion validity of this device in relation to energy expenditure (left wrist, *r* = 0.86; right wrist, *r* = 0.83) and high reliability (intra-individual variance 1.4%, inter-individual variance 2.1%) have been reported previously [43]. A minimum of 6 days including weekends is recommended to capture habitual PA which was within the scope of our fieldwork. Finally, we implemented a robust methodology for data extraction and summary of the data recorded by the GENEactiv device. Reference details [44–49] are given in supplementary material.

In conclusion, we have shown that higher levels of objectively measured PA are associated with reduced adiposity, increased muscle mass, function and decreased risk of sarcopenia. Our results contribute to better understanding of the consequences of different levels of PA and sedentary behaviour among older people. These results suggest that incremental elevations in habitual physical activity in older people may help to decelerate age-related declines in musculoskeletal fitness.

## Electronic supplementary material

Below is the link to the electronic supplementary material.

Supplementary material 1 (DOCX 29 KB)
