# Supplementary material for: Associations Between Objectively Measured Physical Activity, Body Composition and Sarcopenia: Findings from the Hertfordshire Sarcopenia Study (HSS)
Source: Calcif Tissue Int. 2018 Mar 27;103(3):237–45. doi: 10.1007/s00223-018-0413-5 (PMC6049619; doi:10.1007/s00223-018-0413-5)
Supplement: Supplementary file 1 — Supplementary material 1 (DOCX 29 KB) [file 223_2018_413_MOESM1_ESM.docx]

**Supplementary information**

**Use of two recording frequencies**

Originally, participants were asked to activate and wear the device received by post which would then record physical activity for 7 days at 100Hz. However, some participants failed to activate the device so devices were subsequently activated and then posted to participants; the frequency was reduced to 25Hz to enable the device to capture the activity over this longer duration. This is unlikely to have influenced the results as a previous study has shown that out of a range of sampling frequencies, frequencies greater than 10Hz were not associated with greater activity classification accuracy (44). In addition, there was no difference in mean daily acceleration between recordings at 25Hz and 100Hz (p=0.987).

**Processing of accelerometry data**

Initial stages of data processing included estimating and accounting for calibration error (45) and detecting periods of non-wear. Non-wear time was determined using the range and standard deviation of each accelerometer axis using 1 hour windows and moving increments of 15 minutes.

Average daily acceleration over each 24 hour period (in milli-g) was calculated using the gravity subtracted Euclidean norm $(\sqrt{x^{2}+y^{2}+z^{2}}-g;acceleration axes:x,y and z)$ with negative values replaced with zeros (46). Time windows where the range was less than 50 milli-g or the standard deviation was less than 13 milli-g for at least two of the axes were regarded as non-wear periods. One hour time windows ensured that periods of sleep and rest were not categorized as non-wear (45, 47). A similar method of non-wear detection has been used in other studies (47-49) and has been described in detail previously (46). Invalid 15 minute intervals of non-wear were imputed with the average acceleration at similar times on the other days of measurement (46). To ensure that only acceleration due to human movement was recorded, the signal was passed through a frequency filter with a lower and upper bound of 0.2Hz and 15Hz respectively.

## As well as average daily acceleration, summary measures of physical activity included average daily time spent in MVPA (≥100 milli-g) and in non-sedentary physical activity levels (≥40 milli-g). These measures were derived using 5 second epochs and 5 minute bouts. For bouts to be classed as MVPA or non-sedentary, they had to start with an epoch value greater than 100 milli-g or 40 milli-g respectively, and have 80% of succeeding entries larger than the corresponding threshold.
